# Exosome‐derived circTFDP2 promotes prostate cancer progression by preventing PARP1 from caspase‐3‐dependent cleavage

**DOI:** 10.1002/ctm2.1156

**Published:** 2023-01-03

**Authors:** Lifeng Ding, Qiming Zheng, Yudong Lin, Ruyue Wang, Huan Wang, Wenqin Luo, Zeyi Lu, Haiyun Xie, Liangliang Ren, Haohua Lu, Chenhao Yu, Jixuan Zhang, Danyang Shen, Sheng Cheng, Liqun Xia, Gonghui Li, Dingwei Xue

**Affiliations:** ^1^ Department of Urology Sir Run Run Shaw Hospital Zhejiang University School of Medicine Hangzhou China; ^2^ Department of General Surgery The First Affiliated Hospital of Soochow University Suzhou China

**Keywords:** circTFDP2, DNA damage, PARP1, prostate cancer

## Abstract

**Background:**

Circular RNAs (circRNAs) have been reported to play a significant role in tumorigenesis. However, the detailed function of circRNA in prostate cancer (PCa) is still largely unknown.

**Methods:**

We quantified circTFDP2 expression in PCa tissues and adjacent normal tissues using quantitative reverse transcription‐polymerase chain reaction (qRT‐PCR). Colony formation, Cell Counting Kit‐8 (CCK‐8), flow cytometry, transwell, and in vivo progression and metastasis assays were applied to reveal the proliferation and metastatic abilities of circTFDP2 in PCa cells. Mass spectrometry, RNA pulldown, RNA‐immunoprecipitation (RIP), western blotting and immunofluorescence were used for the mechanistic studies. qRT‐PCR and RIP assays were used to explore the regulatory role of eIF4A3 in the biogenesis of circTFDP2. Finally, functional assays showed the effect of circTFDP2‐containing exosomes on PCa cell progression.

**Results:**

circTFDP2 was upregulated in PCa tissues compared with adjacent normal tissues. Furthermore, high circTFDP2 expression was positively correlated with the Gleason score. Functionally, circTFDP2 promoted PCa cell proliferation and metastasis both in vivo and in vitro. Mechanistically, circTFDP2 interacted with poly(ADP‐ribose) polymerase 1 (PARP1) protein in its DNA‐binding domain to prevent it from active caspase‐3‐dependent cleavage, and finally relieved PCa cells from DNA damage. In addition, RNA‐binding protein eIF4A3 can interact with the flanking region of circTFDP2 and promote the biogenesis of circTFDP2. Moreover, exosome‐derived circTFDP2 promoted PCa cell progression.

**Conclusions:**

In general, our study demonstrated that circTFDP2 promoted PCa cell progression through the PARP1/DNA damage axis, which may be a promising therapeutic target for PCa.

## Graphical abstract

1. circTFDP2 is upregulated in prostate cancer tissues and positively correlated with the Gleason score, metastasis status and T stage of prostate cancer patients.

2. circTFDP2 promotes prostate cancer progression via directly binding to PARP1.

3. circTFDP2 could be secreted to the cell culture by exosomes.

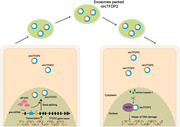

## 1 BACKGROUND

In the western world, prostate cancer (PCa) is the most common solid‐organ malignancy diagnosed in men.^1^, ^2^ Prostate‐specific antigen (PSA) measurement is the most well‐known screening test for PCa. However, PSA screening usually results in the overdiagnosis and overtreatment of patients.^3^, ^4^ Clinical management of PCa patients needs to consider various factors, including stages, histopathology, molecular features and patient backgrounds.^5^ Nevertheless, approximately 30% of PCa cases eventually progress to metastatic PCa, despite the recent progression.^6^ Hence, there is an urgent need to explore the detailed mechanisms underlying the development of PCa.

Circular RNAs (circRNAs) are a new class of noncoding RNAs formed by the back‐splicing of pre‐mRNAs,^7^, ^8^ circRNAs did not get much attention until the advancement of next‐generation sequencing. Several circRNAs have been identified and found to play vital roles in various physiological processes.^9^ Previous research has revealed that circRNAs can exert their functions by binding and sequestrating microRNAs (miRNAs).^10^ Further studies have also demonstrated that circRNAs can interact with RNA‐binding proteins (RBPs), functioning as protein sponges.^11^ Recent research has revealed that circRNAs can encode unique peptides through cap‐independent or m^6^A‐dependent translation.^12^ Functionally, circRNAs were involved in various physiological processes, for instance, maintaining stem‐cell pluripotency, controlling cell differentiation, regulating cell cycle and apoptosis, and angiogenesis.^13^, ^14^, ^15^ However, circRNA functions in PCa have remained poorly understood.

Exosomes are nanosized (30–150 nm) extracellular vesicles surrounded by a lipid bilayer membrane.^16^ They are generated by the endosomal pathway and can be released by most cell types into bodily fluids.^17^ Exosomal components, including proteins, DNA, mRNA, miRNA and circRNAs, can mediate cellular communication. As a result, they can regulate the recipient cells’ gene expression. Hence, exosomes participate in various pathological processes, cardiovascular disease, regeneration disease and cancer, for example.^18^, ^19^, ^20^ Recent studies have demonstrated that exosome‐derived circRNAs have a key role in disease progression, which could be the potential candidate for disease therapy.^21^ However, little research has focused on the specific function of exosome‐derived circRNAs in PCa.

In this work, we reported a circRNA, termed circTFDP2, that was highly expressed in PCa tissues. Functionally, circTFDP2 promoted PCa cell proliferation and metastasis both in vitro and in vivo. Mechanistically, circTFDP2 interacted with poly(ADP‐ribose) polymerase 1 (PARP1) and prevented it from caspase‐3‐dependent cleavage. Moreover, exosomal circTFDP2 promoted PCa cell progression. Notably, our study identified a novel circRNA that prevented PCa cells from DNA damage by preventing PARP1 from caspase‐3‐dependent cleavage, which may be the hopeful therapeutic target for PCa.

## 2 MATERIALS AND METHODS

### 2.1 In vivo tumorigenesis and metastasis assay

For xenograft animal model, 4‐week‐old BALB/c nude mice were used. A total of 10^7^ circTFDP2 stably knockdown or overexpressing or negative control 22Rv‐1 cells were subcutaneously injected into the BALB/c nude mice in 100 μl of 1x phosphate‐buffered saline (PBS). After 6–7 weeks, the tumours were harvested and the width (*a*) and length (*b*) of tumours were measured. The volume was calculated using the formula: *V* = 1/2*ab* ^2^.

For the tail vein metastasis model, 10^7^ circTFDP2 stably knockdown or overexpressing or negative control 22Rv‐1 cells were injected via the tail vein into the 4‐week‐old BALB/c nude mice. After 6–8 weeks, mice were anesthetised, following which the metastatic loci were photographed using in vivo imaging system.

All procedures involving animals were approved by the Ethics Committee of Sir Run Run Shaw Hospital, School of Medicine, Zhejiang University.

### 2.2 RNA pulldown assay

The biotin‐labelled circTFDP2 probe and four biotin‐labelled circTFDP2 segment probes were synthesised by Tsingke (Beijing, China). A total of 2 × 10^7^ 22Rv‐1 and C4‐2B cells were lysed with lysis buffer (50 mM Tris–HCl, pH 7.4, 150 mM NaCl, 2 mM MgCl_2_, 1% NP40, protease inhibitors and RNase inhibitors) at 4°C for 30 min. After centrifugation, the supernatants were incubated with the corresponding probe for 30 min at 4°C, followed by incubation with 50 μl of streptavidin C1 magnetic beads (Invitrogen, USA). The beads were washed 10 times with washing buffer (50 mM Tris–HCl, pH 7.4, 150 mM NaCl, 2 mM MgCl_2_ and 1% NP40), followed by western blotting detection. The biotin‐labelled circTFDP2 probe and four biotin‐labelled circTFDP2 segment probes sequences are listed in Table S4.

### 2.3 Statistical analysis

GraphPad Prism software v8.0 was used for analyses. Data were presented as mean ± SD.

The data were analysed using the *t*‐test for two independent samples or one‐way ANOVA followed by Student–Newman–Keuls test for more than two samples. The correlation analysis between eIF4A3 and circTFDP2 was examined by Pearson's correlation test. All experiments were repeated three times. A *p*‐value less than .05 was considered significant; ^*^ *p* < .05; ^**^ *p* < .01; ^***^ *p* < .001.

Additional method information is listed in Supporting Information.

## 3 RESULTS

### 3.1 circTFDP2 is highly expressed in PCa

**FIGURE 1:**
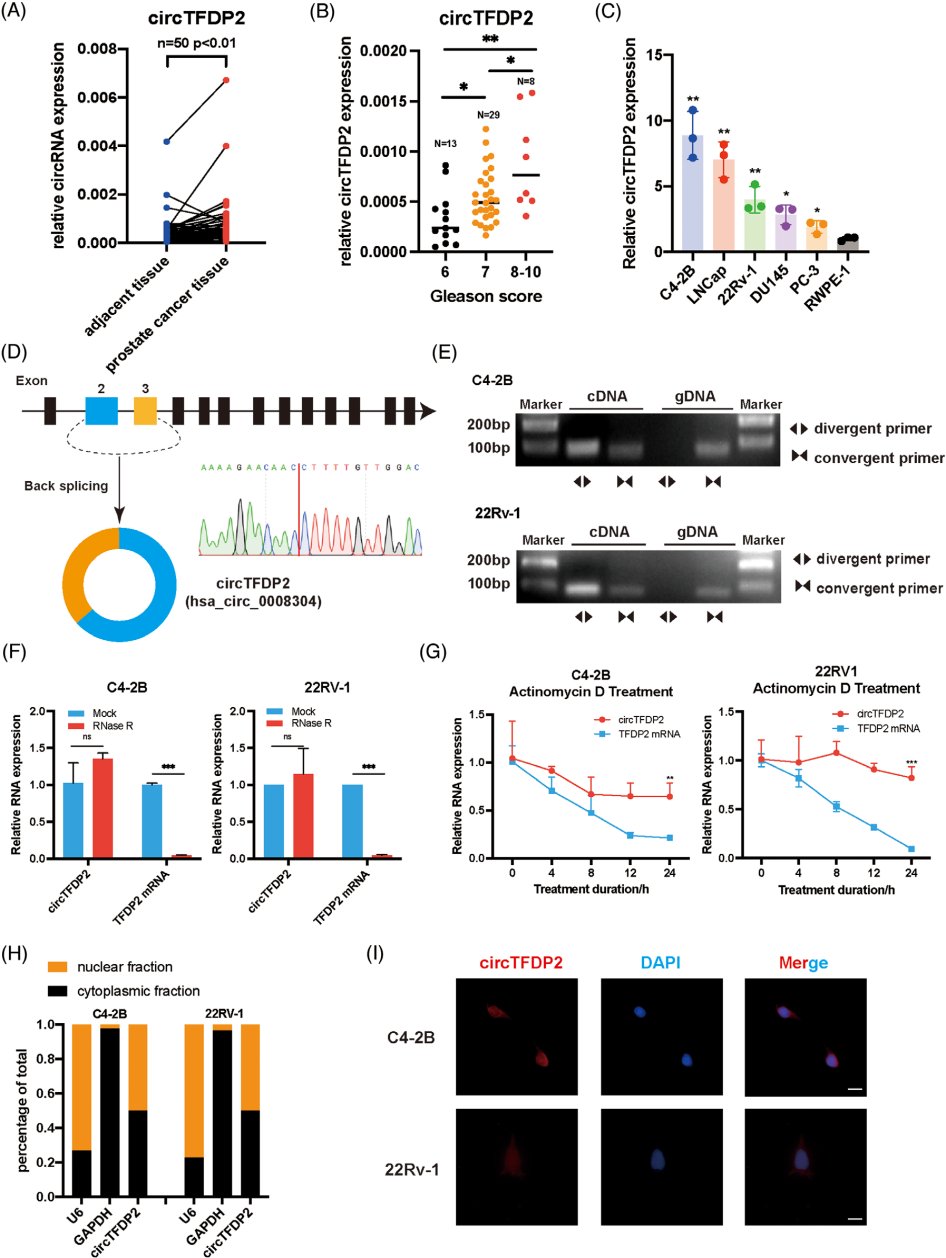
circTFDP2 is upregulated in prostate cancer (PCa). (A) Relative expression of circTFDP2 in 50 paired PCa tissues using qRT‐PCR. (B) Relative expression of circTFDP2 in 50 paired PCa tissues with different Gleason scores using qRT‐PCR. (C) Relative expression of circTFDP2 in PCa cell lines and normal prostate epithelial cell lines using qRT‐PCR. (D) Genomic location of circTFDP2 in PCa and the junction site was detected using Sanger sequencing. (E) PCR of gDNA and cDNA using divergent and convergent primers. (F) Analysis of circTFDP2 and TFDP2 mRNA expression after RNase R treatment in PCa cells. (G) Analysis of circTFDP2 and TFDP2 mRNA expression after actinomycin D treatment at the indicated times. (H) Analysis of circTFDP2 expression in nuclear and cytoplasmic fractions using qRT‐PCR. (I) Fluorescence in situ hybridisation (FISH) assay showing the cellular distribution of circTFDP2 in PCa cells. Scale bar = 10 μm. Data represent mean ± SD from three independent experiments. ^*^ *p* < .05; ^**^ *p* < .01; ^***^ *p* < .001

To identify the functional circRNAs in PCa, we reanalysed our circRNA array data.^22^ As a result, 27 differentially expressed circRNAs were discovered. Among these, 19 circRNAs were selected that could be found in circBase database. Subsequently, the expression of these circRNAs was verified in 50 paired PCa tumour and adjacent normal specimens using qRT‐PCR. Surprisingly, hsa_circ_0008304, which was derived from TFDP2 and designated as circTFDP2, was significantly upregulated in PCa samples (Figure 1A). Meanwhile, circTFDP2 expression was positively correlated with the Gleason score (Figure 1B). Also, circTFDP2 expression was highly expressed in PCa patients with metastatic foci (Figure S1A). Compared with patients with T2 stage PCa, those with T4 stage PCa exhibited higher circTFDP2 expression (Figure S1B). Moreover, compared with RWPE‐1, the PCa cell lines exhibited an upregulation of circTFDP2 (Figure 1C). These data confirm that circTFDP2 is upregulated in PCa.

circTFDP2 was derived from exons 2–3 of the TFDP2 by back‐splicing, and the junction site was confirmed through Sanger sequencing (Figure 1D). Using cDNA and genomic DNA (gDNA), circTFDP2 was found to be amplified only from cDNA using divergent primers, while TFDP2 mRNA could be reversed from both cDNA and gDNA using convergent primers (Figure 1E). We also observed that circTFDP2 was more resistant to RNase R and actinomycin D treatments than the linear transcript (Figure 1F,G). Moreover, the nuclear and cytoplasmic fractionation assay confirmed that circTFDP2 was located in both nuclear and cytoplasmic fractions (Figure 1H). RNA fluorescence in situ hybridisation (FISH) assay also received the same results using Cy3‐labelled circTFDP2‐specific probe that targeted the junction site (Figure 1I). These data confirm that circTFDP2 is a circRNA.

### 3.2 eIF4A3 regulates circTFDP2 biogenesis in PCa

**FIGURE 2:**
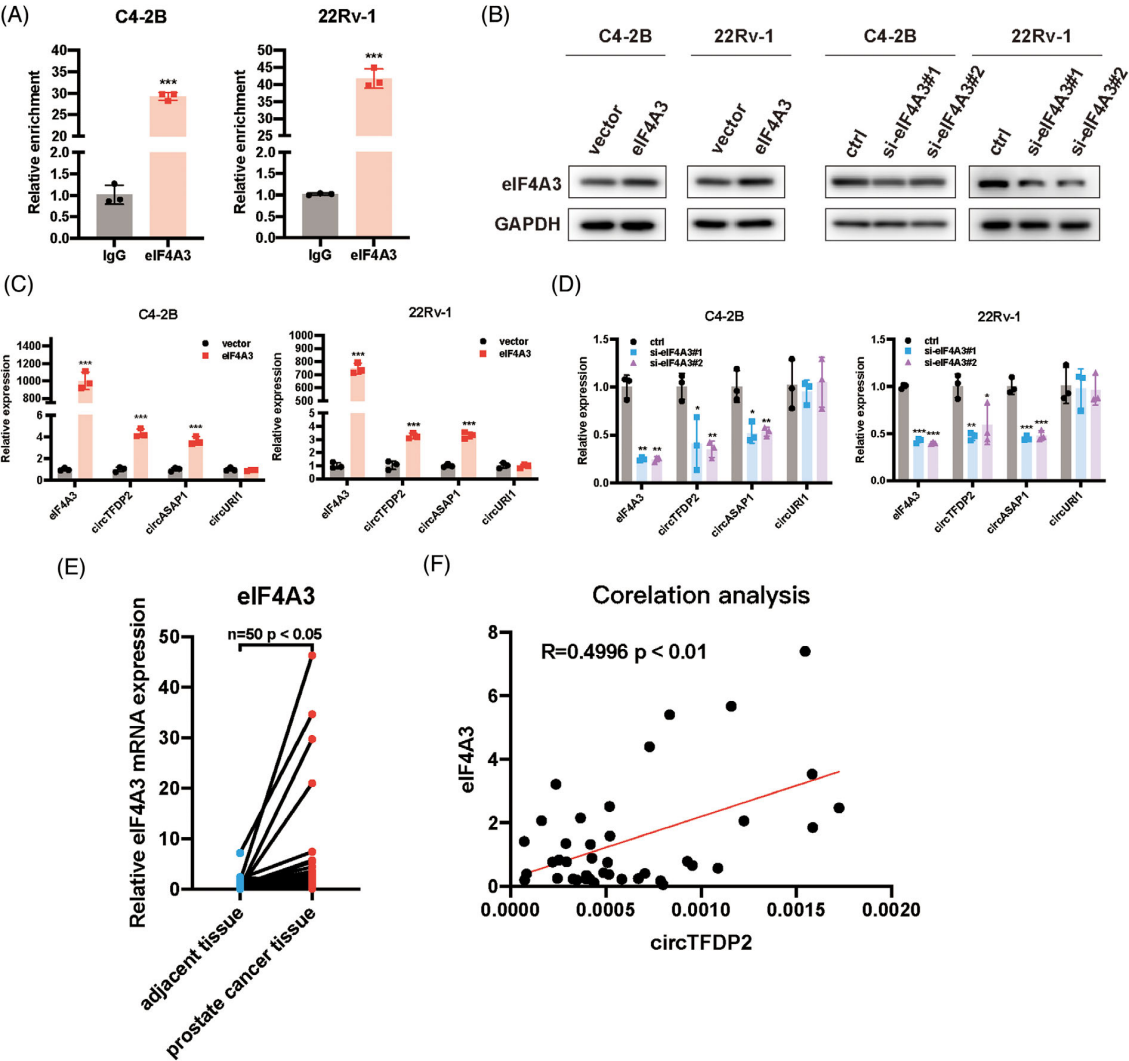
eIF4A3 regulated the biogenesis of circTFDP2. (A) eIF4A3 RNA‐immunoprecipitation (RIP) assay detected the interaction between eIF4A3 and flanking sequence of circTFDP2. (B) Western blotting assay showing the overexpression and knockdown efficiency of eIF4A3. (C) Analysis of circTFDP2 expression with eIF4A3 overexpression. (D) Analysis of circTFDP2 expression with eIF4A3 knockdown. (E) Relative expression of eIF4A3 in 50 paired prostate cancer (PCa) tissues using qRT‐PCR. (F) Correlation analysis of the expression between eIF4A3 and circTFDP2 in 50 PCa specimens. Data represent mean ± SD from three independent experiments. ^*^ *p* < .05; ^**^ *p* < .01; ^***^ *p* < .001

Previous research has shown that several RBPs can regulate circRNA biogenesis.^7^, ^23^ Therefore, the potential RBP sites at the flanking region of circTFDP2 and two putative eIF4A3‐binding sites in the flanking region of circTFDP2 were identified using the CircInteractome database (https://circinteractome.nia.nih.gov/) (Figure S2A). Then, RNA‐immunoprecipitation (RIP) assay revealed that eIF4A3 can bind to the flanking region of circTFDP2 but not to the negative control antibody immunoglobulin G (IgG) (Figure 2A). Therefore, we hypothesised that eIF4A3 controlled circTFDP2 biogenesis. Then, eIF4A3 siRNAs or plasmids were transfected into the PCa cells, and the transfection efficiency was detected (Figure 2B). Furthermore, qRT‐PCR assay was applied to explore the role of eIF4A3 in circTFDP2 expression. And circASAP1, which has eIF4A3‐binding sites in the flanking region, was used as a positive control, whereas circURI, which does not have eIF4A3‐binding sites, was used as a negative control. The result demonstrated that eIF4A3 overexpression enhanced circTFDP2 expression, while eIF4A3 knockdown decreased its expression (Figure 2C,D). Next, we detected eIF4A3 expression in 50 paired PCa tissues, and the results demonstrated that compared with normal tissues, eIF4A3 was highly expressed in PCa tissues (Figure 2E). Correlation analysis demonstrated that eIF4A3 expression was positively correlated with circTFDP2 expression in PCa tissues (Figure 2F). In conclusion, these data reveal that eIF4A3 regulates the generation of circTFDP2 in PCa tissues.

### 3.3 circTFDP2 promotes PCa cell proliferation and inhibits their apoptosis

**FIGURE 3:**
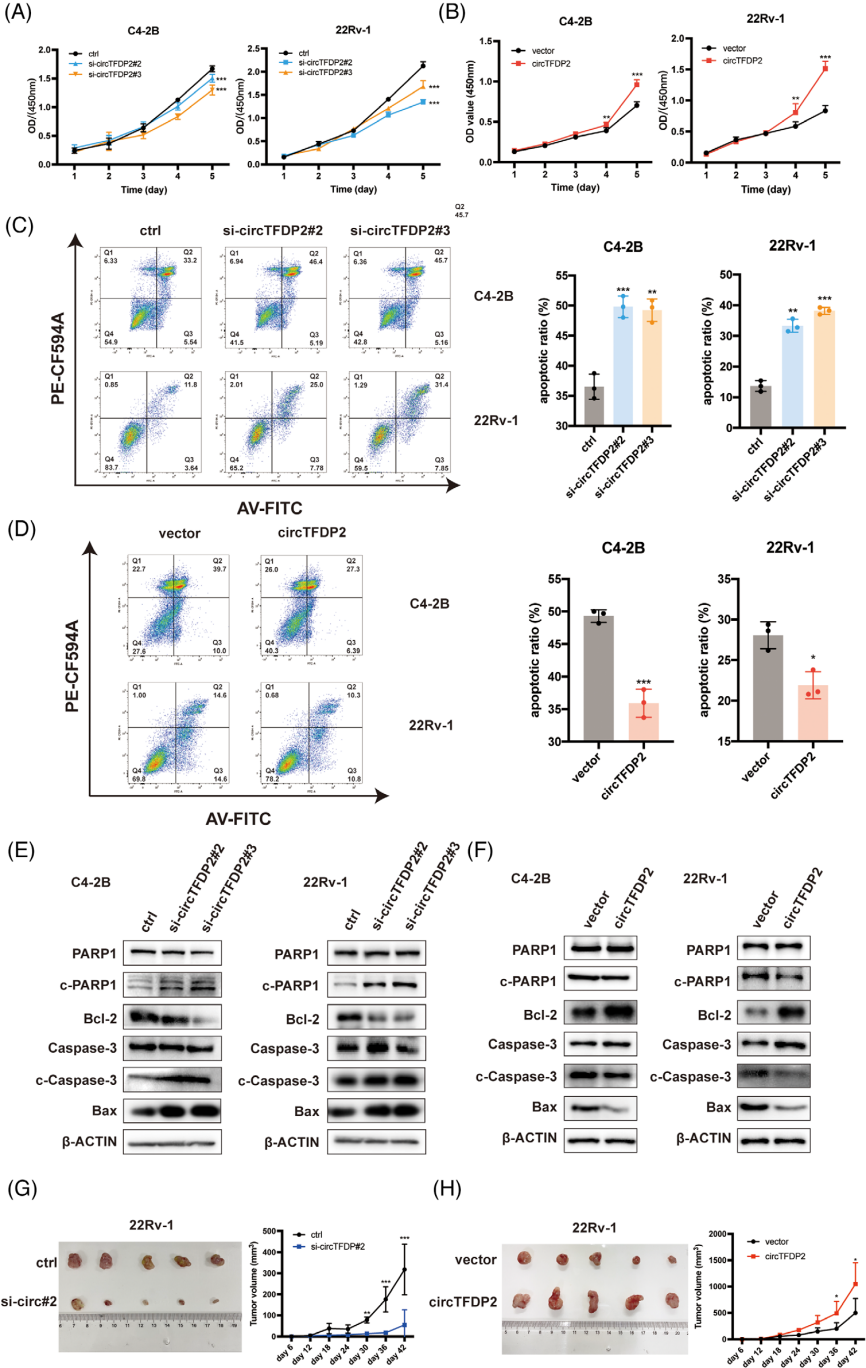
circTFDP2 promoted prostate cancer (PCa) proliferation and inhibits PCa apoptosis. (A) Cell Counting Kit‐8 (CCK‐8) assay of C4‐2B and 22Rv‐1 cells with circTFDP2 knockdown. (B) CCK‐8 assay of C4‐2B and 22Rv‐1 cells with circTFDP2 overexpression. (C) Left: apoptotic analysis by flow cytometry; right: apoptotic ratio of C4‐2B and 22Rv‐1 cells with circTFDP2 knockdown. (D) Left: apoptosis analysis by flow cytometry; right: apoptotic ratio of C4‐2B and 22Rv‐1 cells with circTFDP2 overexpression. (E) Protein levels of apoptotic markers in PCa cells with circTFDP2 knockdown. (F) Protein levels of apoptotic markers in PCa cells with circTFDP2 overexpression. (G) Xenograft animal model showing the volume of subcutaneous tumours with circTFDP2 knockdown (*n* = 5 per group). (H) Xenograft animal model showing the volume of subcutaneous tumours with circTFDP2 overexpression (*n* = 5 per group). Data represent mean ± SD from three independent experiments. ^*^ *p* < .05; ^**^ *p* < .01; ^***^ *p* < .001

To explore the specific role of circTFDP2 in PCa, circTFDP2‐specific siRNAs and circTFDP2 overexpression plasmids were designed and transfected into the PCa cells. The knockdown or overexpression efficiency was verified using qRT‐PCR (Figure S3A,B). Cell Counting Kit‐8 (CCK‐8) and colony formation assays revealed that silencing of circTFDP2 markedly impaired the proliferation ability of PCa cells (Figures 3A and S3C), while overexpression of circTFDP2 enhanced the proliferation ability of PCa cells (Figures 3B and S3D). Furthermore, the flow cytometry assay demonstrated that knockdown of circTFDP2 elevated the apoptotic ratio of PCa cells (Figure 3C), whereas circTFDP2 overexpression decreased its apoptotic rate (Figure 3D). Next, we detected the apoptosis‐related markers using western blotting. The results demonstrated that circTFDP2 knockdown increased the expression of cleaved caspase‐3, cleaved PARP1 and Bax, and decreased Bcl‐2 expression (Figure 3E), and circTFDP2 overexpression decreased the expression of cleaved caspase‐3, cleaved PARP1 and Bax, and increased Bcl‐2 expression (Figure 3F). Later, we detected the oncogenic role of circTFDP2 in vivo by establishing a xenograft tumour model in nude mice. The results indicated that circTFDP2 knockdown markedly inhibited tumour growth in vivo (Figure 3G). Conversely, overexpression of circTFDP2 increased PCa cell growth in vivo (Figure 3H). The results demonstrate that circTFDP2 promotes PCa cell proliferation.

### 3.4 circTFDP2 promotes PCa cell migration

**FIGURE 4:**
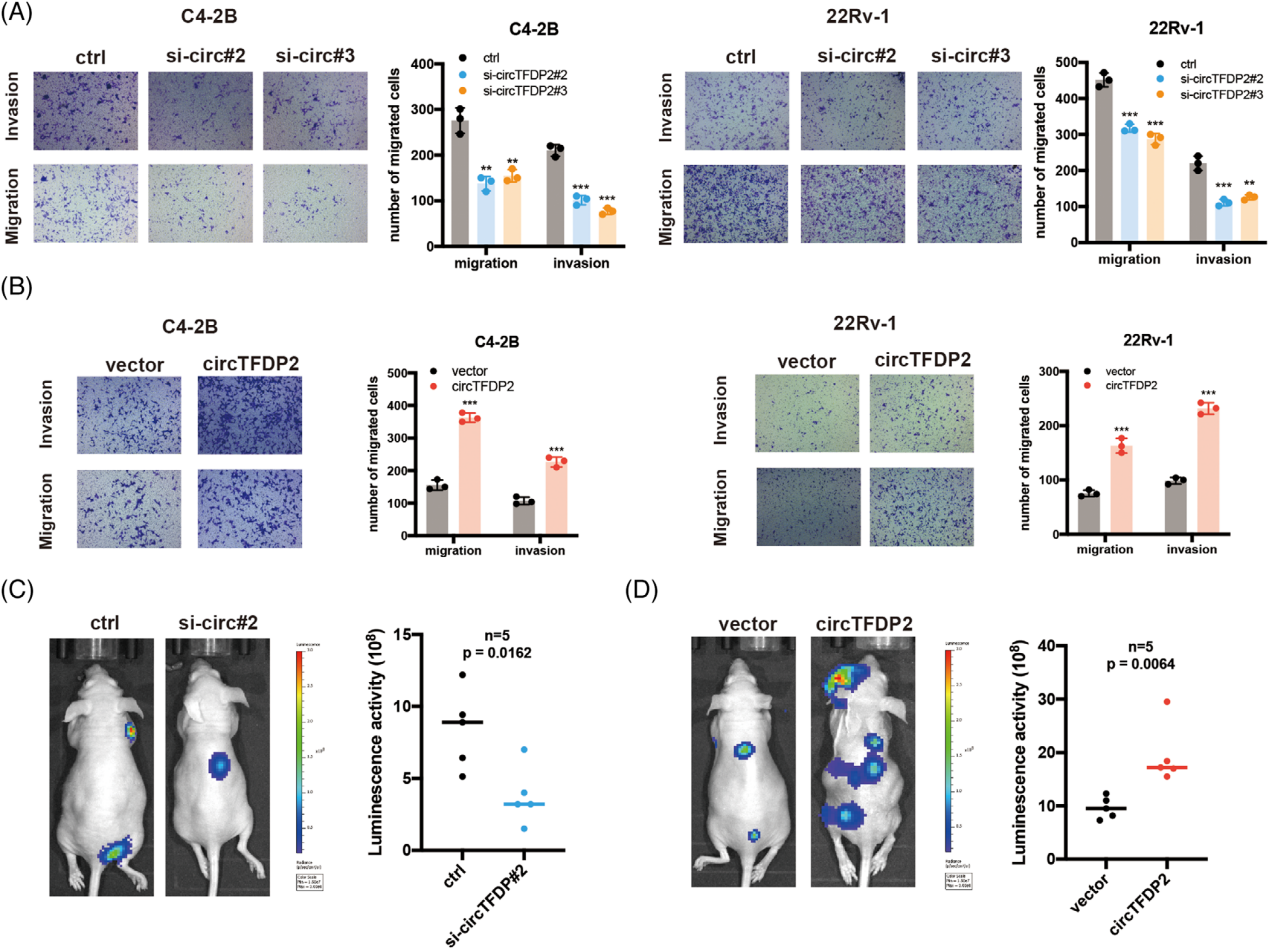
circTFDP2 promoted prostate cancer (PCa) metastasis both in vitro and in vivo. (A) Transwell assay for C4‐2B and 22Rv‐1 cells with circTFDP2 knockdown. (B) Transwell assay for C4‐2B and 22Rv‐1 cells with circTFDP2 overexpression. (C) Tail vein metastasis model for 22Rv‐1 cells with circTFDP2 knockdown (*n* = 5 per group). (D) Tail vein metastasis model for 22Rv‐1 cells with circTFDP2 overexpression (*n* = 5 per group). Data represent mean ± SD from three independent experiments. ^*^ *p* < .05; ^**^ *p* < .01; ^***^ *p* < .001

Subsequently, we evaluated the function of circTFDP2 in PCa cell metastasis. Transwell assays revealed that circTFDP2 knockdown significantly reduced the invasion and migration abilities of PCa cells (Figure 4A), whereas its overexpression significantly enhanced these abilities (Figure 4B). Moreover, the nude mouse metastasis models showed that circTFDP2 knockdown inhibited the metastatic ability of 22Rv‐1 cells in vivo (Figure 4C), while circTFDP2 overexpression promoted 22Rv1 cells’ metastatic ability in vivo (Figure 4D). These data demonstrate that circTFDP2 promotes PCa cell metastasis.

### 3.5 circTFDP2 interacts with PARP1 and prevents it from cleavage

**FIGURE 5:**
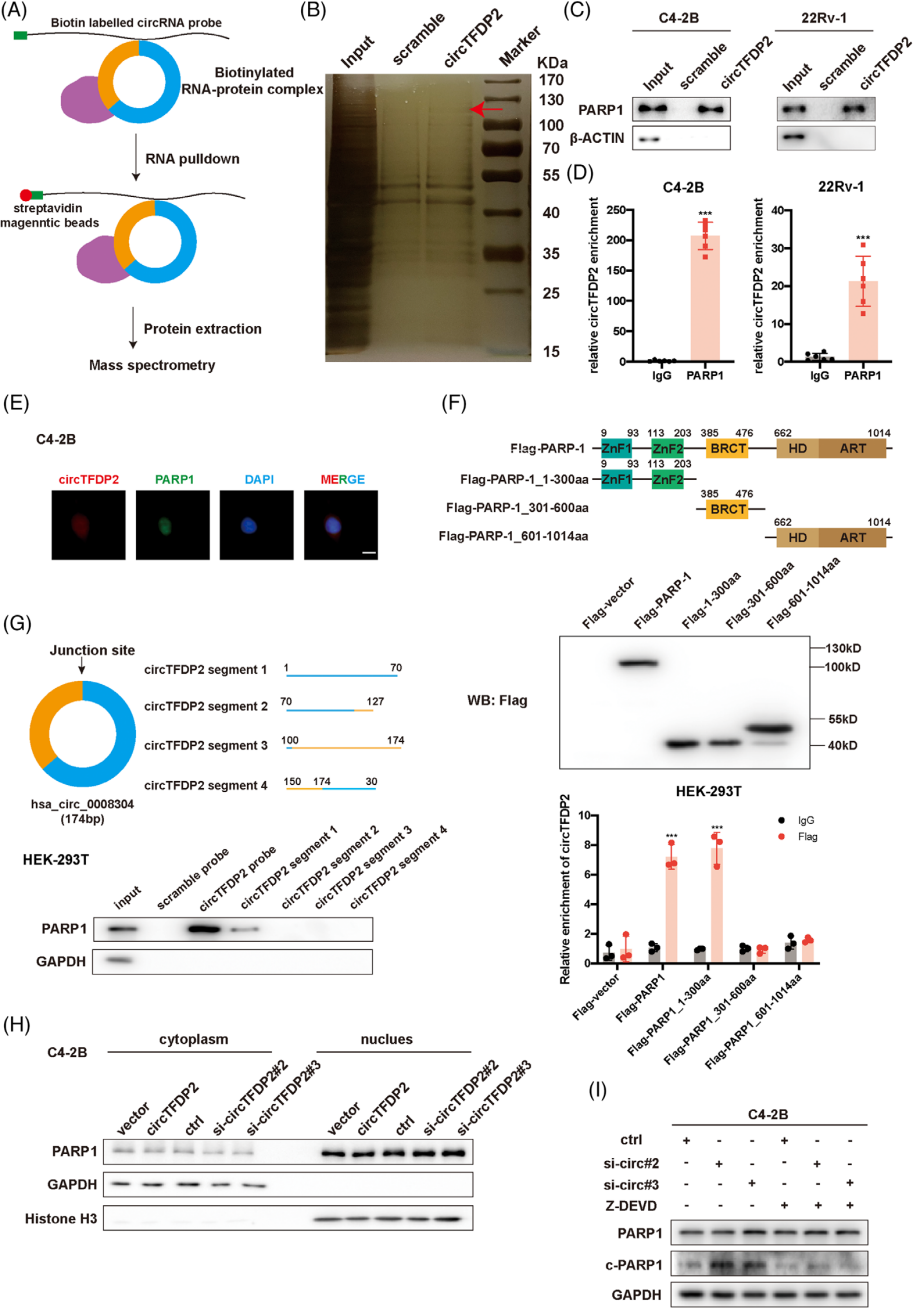
circTFDP2 physically interacted with poly(ADP‐ribose) polymerase 1 (PARP1). (A) Schematic diagram showing the processes of RNA pulldown followed by mass spectrometry. (B) Silver staining showing the circTFDP2‐binding proteins. (C) RNA pulldown assay detected the interaction between circTFDP2 and PARP1. (D) PARP1‐RNA‐immunoprecipitation (RIP) assay detected the interaction between PARP1 and circTFDP2. (E) Immunofluorescence (IF) and fluorescence in situ hybridisation (FISH) assays showing the co‐localisation of circTFDP2 and PARP1. Scale bar = 10 μm. (F) Upper: schematic diagram showing the truncations of flag‐tagged PARP1 proteins; middle: western blotting showing the verification of truncations of flag‐tagged PARP1 proteins; bottom: flag‐RIP assay showing the binding capacity of circTFDP2 and truncations of flag‐tagged PARP1 proteins. (G) Upper: schematic diagram showing the truncation of biotin‐labelled circTFDP2; bottom: RNA pulldown assay showing the binding capacity of PARP1 and truncation of biotin‐labelled circTFDP2. (H) Cellular distribution of PARP1 in prostate cancer (PCa) cells with circTFDP2 knockdown and overexpression. (I) Protein levels of cPARP1 in PCa cells with circTFDP2 knockdown or Z‐DEVD‐FMK added. Data represent mean ± SD from three independent experiments. ^*^ *p* < .05; ^**^ *p* < .01; ^***^ *p* < .001

To identify the molecular mechanism by which circTFDP2 regulates PCa progression, we first analysed the circRNADb database (http://reprod.njmu.edu.cn/cgi‐bin/circrnadb/circRNADb.php). The results showed that circTFDP2 did not contain an internal ribosomal entry site (IRES) or open reading frame (ORF) region, suggesting that circTFDP2 had lower protein‐coding potential (Figure S4A). Subsequently, since circTFDP2 was located in both the nuclear and cytoplasmic fractions, we examined whether circTFDP2 can function as an miRNA sponge. AGO2‐RIP assays demonstrated that circTFDP2 cannot interact with the AGO2 protein, suggesting its inability to serve as an miRNA sponge (Figure S4B). Next, we designed a specific biotin‐labelled circTFDP2 probe to explore its protein‐binding role in PCa cells. RNA pulldown assay followed by silver staining and mass spectrometry (MS) analysis were performed for this analysis (Figure 5A,B), and 26 proteins were found to specifically interact with circTFDP2 (Table S6). Among these RBPs, PARP1 was selected as the potential downstream target, for which PARP1 participates in the progression of castration‐resistant prostate cancer.^24^, ^25^ Next, RNA pulldown assays followed by western blotting were performed to validate the MS data (Figure 5C). Meanwhile, PARP1‐RIP assay also confirmed the interaction between PARP1 and circTFDP2 (Figure 5D). Finally, FISH and immunofluorescence (IF) assays identified the co‐localisation between PARP1 and circTFDP2 (Figure 5E). These data indicate that circTFDP2 and PARP1 form the RNA–protein complex in PCa cells.

Then, we constructed three flag‐tagged PARP1 truncations to identify the PARP1 domain that determined the interaction between PARP1 and circTFDP2. Flag‐RIP assay result showed that the first region (DNA‐binding region) was crucial for the binding between PARP1 and circTFDP2 (Figure 5F). Subsequently, based on the prediction result of the catRAPID algorithm (Figure S4C), four biotin‐labelled circTFDP2 segment probes were designed. The RNA pulldown assay result suggested that the segment 1 of circTFDP2 was indispensable for the interaction between PARP1 and circTFDP2 (Figure 5G). These results confirm the interaction between the circTFDP2 and PARP1.

Based on the interaction between PARP1 and circTFDP2, we examined whether PARP1 affected circTFDP2 expression. The results showed that PARP1 had little effect on circTFDP2 expression in PCa cells (Figure S4D). Moreover, overexpression or knockdown of circTFDP2 failed to alter PARP1 expression (Figure 3E,F), suggesting that circTFDP2 did not regulate PARP1 expression at the posttranscriptional level. Moreover, changing the expression of circTFDP2 failed to alter the cellular localisation of PARP1 (Figure 5H). Since circTFDP2 binds to the DNA‐binding region of PARP1, which was recognised and cleaved by active caspase‐3, we hypothesised that whether circTFDP2 influenced this process. Western blotting assay revealed that overexpression of circTFDP2 decreased the expression of cleaved PARP1, while knockdown of circTFDP2 increased the expression of cleaved PARP1 (Figure 3E,F). Then, western blotting assay also demonstrated that the enhanced PARP1 cleavage in circTFDP2 knockdown PCa cells was blocked by Z‐DEVD‐FMK, a caspase‐3 inhibitor, suggesting that circTFDP2 prevented PARP1 from active caspase‐3‐dependent cleavage (Figure 5I). These data demonstrate that circTFDP2 can attenuate PARP1 cleavage.

### 3.6 PARP1 promotes prostate cancer cell proliferation and migration

**FIGURE 6:**
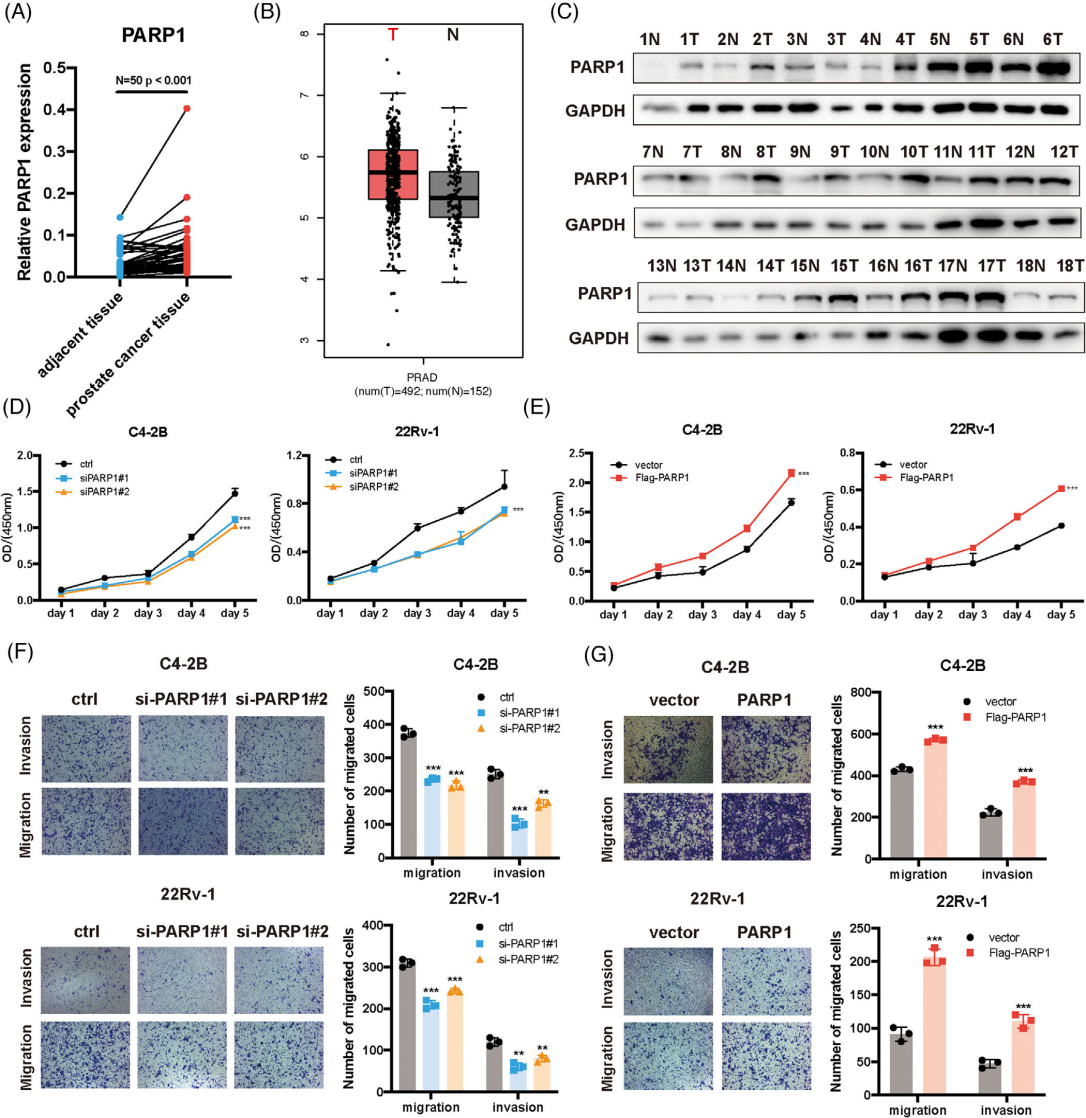
Poly(ADP‐ribose) polymerase 1 (PARP1) promoted prostate cancer (PCa) cell progression. (A) Relative expression of PARP1 in 50 paired PCa tissues using qRT‐PCR. (B) Relative expression of PARP1 in TCGA database. (C) Relative expression of PARP1 protein in 18 paired PCa tissues. (D) Cell Counting Kit‐8 (CCK‐8) assay for C4‐2B and 22Rv‐1 cells with PARP1 knockdown. (E) CCK‐8 assay for C4‐2B and 22Rv‐1 cells with PARP1 overexpression. (F) Transwell assay for C4‐2B and 22Rv‐1 cells with PARP1 knockdown. (G) Transwell assay for C4‐2B and 22Rv‐1 cells with PARP1 overexpression. Data represent mean ± SD from three independent experiments. ^*^ *p* < .05; ^**^ *p* < .01; ^***^ *p* < .001

PARP1 is one of the most critical members of PARP and is involved in many cellular processes, such as stress response, DNA repair and apoptosis.^26^, ^27^ Since circTFDP2 binds to PARP1 and prevents it from cleavage, we examined PARP1 expression in our 50 paired of PCa tumour tissues and adjacent normal specimens using RT‐qPCR. The results revealed that PARP1 was highly expressed in PCa tissues (Figure 6A). After analysis using the TCGA database, the same result was obtained (Figure 6B). Moreover, western blotting assay demonstrated that compared with adjacent normal tissues, PCa tissues exhibited an upregulation of PARP1 at the protein level (Figure 6C). Further, correlation analysis demonstrated that circTFDP2 expression was positively correlated with that of PARP1 (Figure S5A). To explore the function of PARP1 in PCa, CCK‐8 and transwell assays were conducted. CCK‐8 assay showed that knockdown of PARP1 markedly impaired the proliferation ability of PCa cells (Figure 6D), whereas its overexpression enhanced their ability (Figure 6E). Moreover, veliparib, a PARP1 inhibitor, was shown to restrain PCa cell proliferation (Figure S5B). Next, transwell assay revealed that knockdown of PARP1 significantly inhibited the metastatic abilities of PCa cells (Figure 6F), whereas its overexpression promoted their abilities (Figure 6G). Meanwhile, veliparib inhibited PCa cell metastatic ability (Figure S5C). These data demonstrate that PARP1 promotes PCa cells proliferation and migration.

### 3.7 circTFDP2 regulates DNA damage in prostate cancer cells via PARP1

**FIGURE 7:**
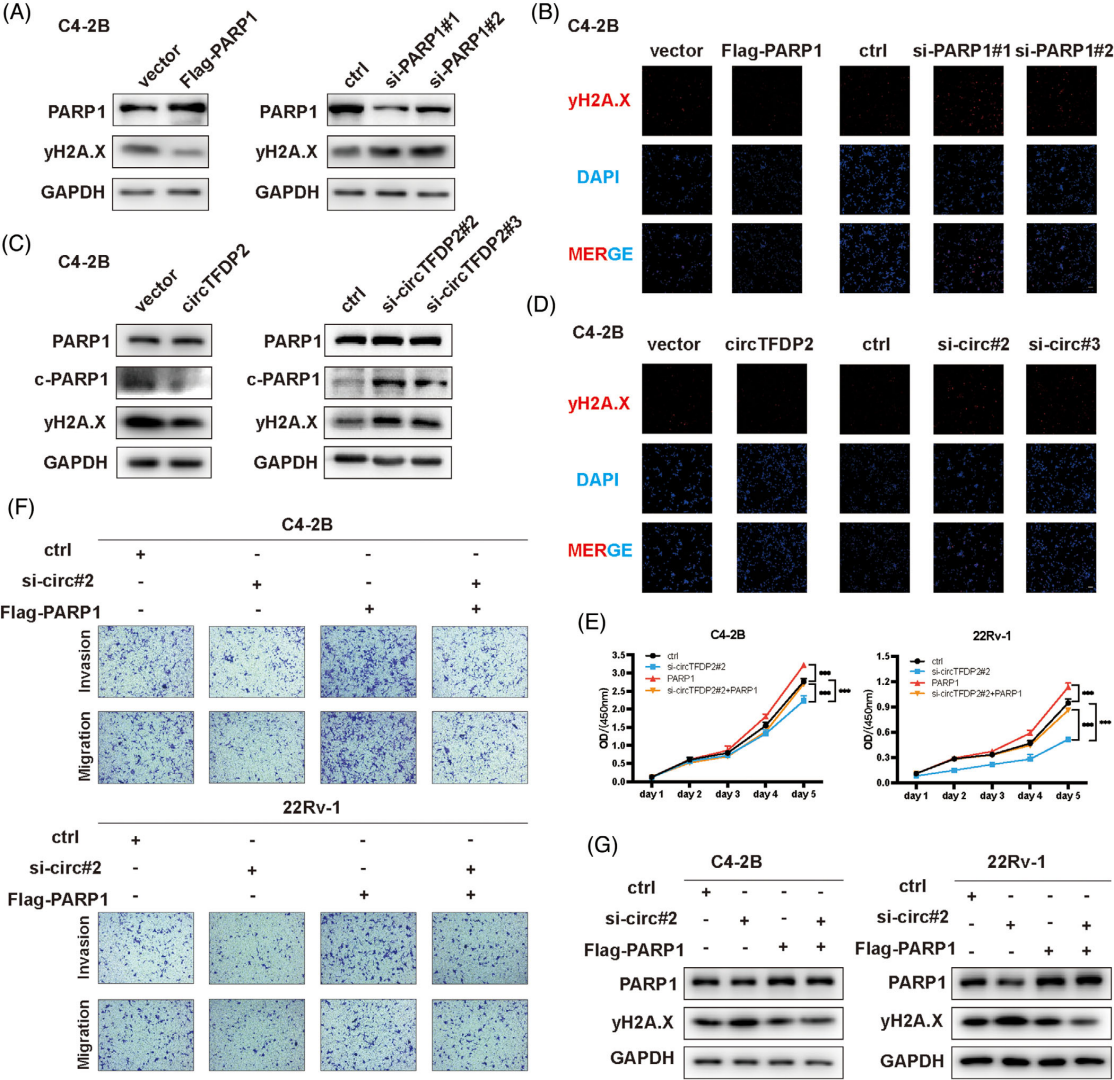
circTFDP2 regulated DNA damage via poly(ADP‐ribose) polymerase 1 (PARP1). (A) Protein levels of DNA damage marker in prostate cancer (PCa) cells with PARP1 overexpression or knockdown. (b) Immunofluorescence (IF) showing the yH2A.X expression with PARP1 overexpression or knockdown. Scale bar = 100 μm. (C) Protein levels of DNA damage marker in PCa cells with circTFDP2 overexpression or knockdown. (D) IF showing the yH2A.X expression with circTFDP2 overexpression or knockdown. Scale bar = 100 μm. (E) Cell Counting Kit‐8 (CCK‐8) assay for C4‐2B and 22Rv‐1 cells with circTFDP2 knockdown or PARP1 overexpression. (F) Transwell assay for C4‐2B and 22Rv‐1 cells with circTFDP2 knockdown or PARP1 overexpression. (G) Protein levels of DNA damage marker with circTFDP2 knockdown or PAPR1 overexpression. Data represent mean ± SD from three independent experiments. ^*^ *p* < .05; ^**^ *p* < .01; ^***^ *p* < .001

Since PARP1 is the vital molecule for single‐strand break repair, we postulated that circTFDP2 might also influence DNA damage repair by preventing PARP1 from cleavage. Western blotting and IF staining were used for the detection of DNA damage marker yH2A.X. As predicted, PARP1 overexpression led to the decrease in yH2A.X in PCa cells, while its knockdown led to an increase in yH2A.X (Figures 7A,B and S6A–C). Meanwhile, we evaluated the role of circTFDP2 in DNA damage. The results revealed that overexpression of circTFDP2 relieved DNA damage in PCa cells, while inhibition of circTFDP2 exacerbated DNA damage (Figures 7C,D and S6D–F), indicating that circTFDP2 was involved in DNA damage of PCa cells.

Subsequently, the CCK‐8 assay revealed that circTFDP2 silencing impaired the PCa cell proliferation ability, whereas the PARP1 overexpression reversed this impairment (Figure 7E). Similarly, the transwell assay demonstrated that PARP1 overexpression could rescue the inhibitory effect of circTFDP2 knockdown (Figures 7F and S6G). These data revealed that circTFDP2 promoted PCa progression via PARP1. Western blotting analysis also showed that the promoting effect of circTFDP2 knockdown on DNA damage could be reversed through overexpression of PARP1 (Figure 7G), indicating the regulatory effect of circTFDP2 on DNA damage via PARP1.

### 3.8 Exosome‐delivered circTFDP2 promotes PCa cell progression

**FIGURE 8:**
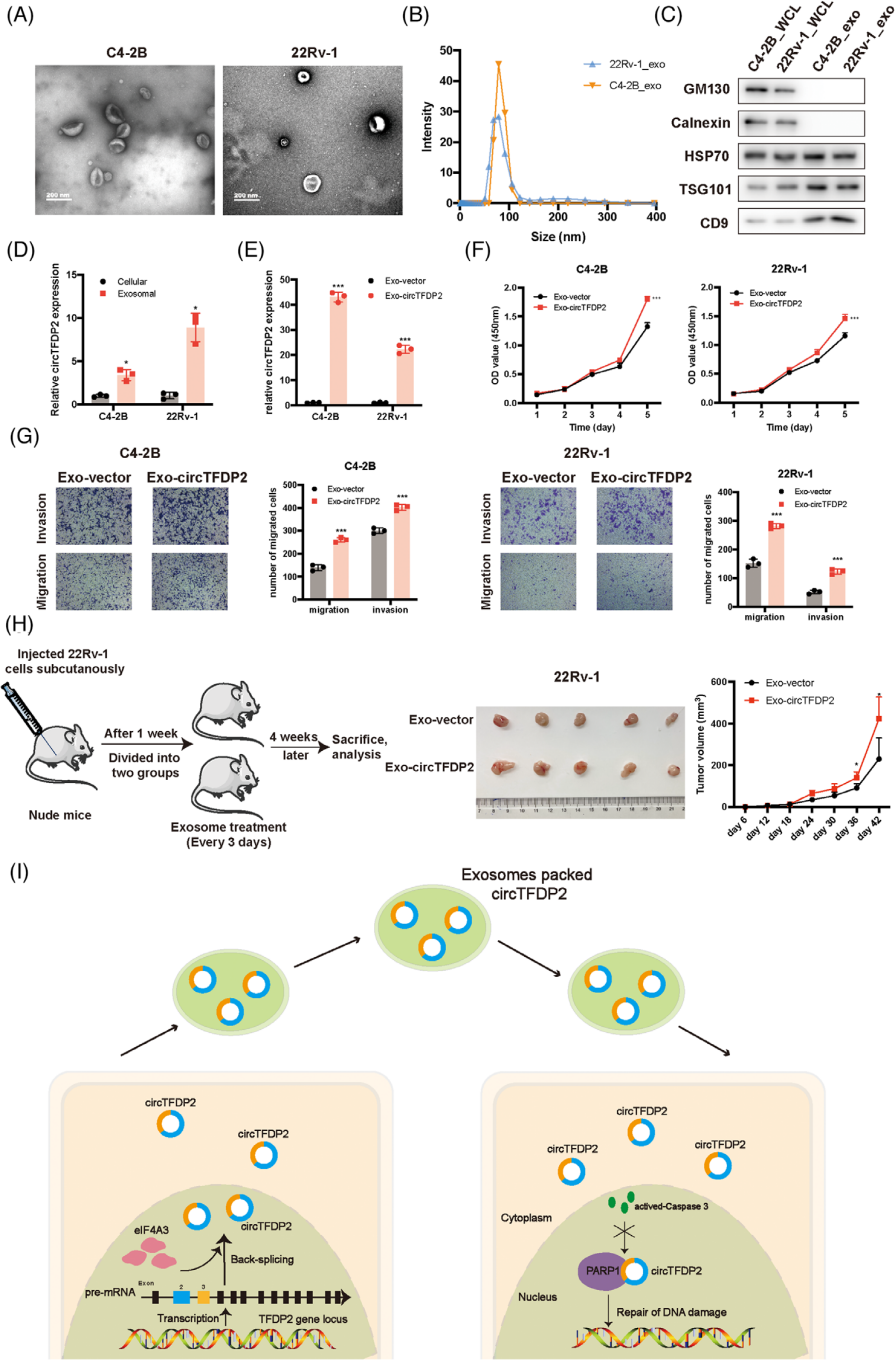
Exosome‐derived circTFDP2 promoted prostate cancer (PCa) progression. (A) Observation of PCa cell‐derived exosomes using transmission electron microscopy. (B) Nanoparticle tracking analysis of exosomes purified from C4‐2B and 22Rv‐1 cells. (C) Western blotting assays showing the presence of exosome markers. (D) Analysis of circTFDP2 expression in cell culture media and cytosol. (E) Analysis of circTFDP2 expression in PCa cells treated with exosomes from circTFDP2 overexpression or vector cells. (F) Cell Counting Kit‐8 (CCK‐8) assay for C4‐2B and 22Rv‐1 cells treated with exosomes from circTFDP2 overexpression or vector cells. (G) Transwell assay for C4‐2B and 22Rv‐1 cells treated with exosomes from circTFDP2 overexpression or vector cells. (H) Left: schematic diagram showing the establishment of xenograft animal model; right: xenograft animal model showing the volume of subcutaneous tumours (*n* = 5 per group). (I) The schematic diagram illustrating the role of circTFDP2 in PCa. ^*^ *p* < .05; ^**^ *p* < .01; ^***^ *p* < .001

Emerging studies have revealed that cancer cell‐secreted exosomes have the crucial roles in the regulation of the intracellular communication, and are thus involved in various biological processes.^28^, ^29^ Since exosomes contain various types of noncoding RNAs, including miRNAs, long noncoding RNAs (lncRNAs) and circRNAs,^30^, ^31^ we wondered whether circTFDP2 is enriched in exosomes. The exosomes were collected and characterised from the cell culture medium of PCa cell lines. Transmission electron microscopy revealed that the exosomes from these two cell lines exhibited the typical rounded shapes (Figure 8A). The nanoparticle tracking analysis showed that the size of these exosomes range from about 60 to 130 nm (Figure 8B). Western blotting assay confirmed the presence of exosome markers HSP70, TSG101 and CD9, while the Golgi apparatus marker GM130 and the endoplasmic reticulum marker calnexin were not found (Figure 8C). These data reveal that exosomes are successfully isolated from the C4‐2B and 22Rv‐1 cell lines.

qPT‐PCR assay showed that exosomes from the cell culture medium contained more circTFDP2 than those in the cytosol (Figure 8D). Furthermore, qPT‐PCR assay revealed that circTFDP2 was more enriched in the exosomes from the culture medium of circTFDP2 overexpressing PCa cells than in those from the culture medium of vector PCa cells (Figure 8E). In order to explored the role of exosome‐delivered circTFDP2 in PCa cell progression, CCK‐8 and transwell assays were conducted. The results demonstrated that the exosomes from the circTFDP2 overexpression cells enhanced PCa cell proliferation and metastasis (Figure 8F,G). Then, the xenograft tumour model also demonstrated that exosomes from circTFDP2 overexpressing cells significantly promoted PCa cells proliferation in vivo (Figure 8H). Taken together, these data show that exosome‐delivered circTFDP2 promotes PCa progression.

## 4 DISCUSSION

Emerging evidence has demonstrated that circRNAs are involved in various diseases, especially in cancer. However, their functions in PCa remain unclear. Yu et al.^32^ reported that hsa_circ_0003258 promoted PCa progression by binding to IGF2BP3, subsequently elevating HDAC4 mRNA stability. Another group revealed that circARHGAP29 enhanced the stability of LDHA mRNA by interacting with IGF2BP2 protein to promote aerobic glycolysis in PCa.^33^ In addition, circRNA FOXO3 enhanced the resistance of PCa cells to docetaxel by increasing the expression of FOXO3.^34^ Similarly, we identified that a novel circRNA, termed as circTFDP2, was highly expressed in PCa tissues compared with adjacent normal specimens. By manipulating the expression of circTFDP2 in PCa cells, we observed that circTFDP2 promoted PCa cell proliferation and metastasis, suggesting that circTFDP2 was an oncogenic circRNA in PCa.

It has been proposed that circRNAs are involved in various physiological processes. The most well‐studied mechanism of circRNAs is their function as miRNA sponges. For example, circRNA_400029 promoted cervical cancer progression by sponging miR‐1285‐3p, finally regulating TLN1 expression.^35^ Moreover, circRNAs can interact with RBPs. Zhang et al.^11^ showed that circVPS13C could bind with RRBP1 proteins to decrease the stability of IFITM1 mRNA, eventually promoting pituitary adenoma progression. Besides, a small subset of circRNAs has been found translatable that contain IRES and ORF. circCHEK1 has been found to encode the peptide circCHEK1_246aa to promote multiple myeloma progression.^36^ Nevertheless, our study found that circTFDP2 did not have an IRES sequence, suggesting that circTFDP2 was untranslatable. Also, AGO2‐RIP assay revealed that circTFDP2 could not serve as an miRNA sponge. Furthermore, RNA pulldown assay followed by MS was performed, and PARP1 was selected as the target protein. Then, protein truncation assay suggested that circTFDP2 interacted with the DNA‐binding domain of PARP1, which included the active caspase‐3 cleavage site. Further studies confirmed that circTFDP2 prevented PARP1 from active caspase‐3‐dependent cleavage.

Many noncoding RNAs have been reported to be involved in DNA damage signalling. For example, lncRNA BGL3 binds to PARP1 and BARD1, resulting in the retention of the PARP1/BARD1 complex in DNA double‐strand sites.^37^ Moreover, lncRNA MALAT1 was also found to interact with PARP1, influencing the DNA damage and apoptosis in multiple myeloma cells.^38^ However, there is no report of PARP1‐related circRNAs so far. In this study, we found that overexpression or knockdown of circTFDP2 relieved or exacerbated the DNA damage in PCa cells and that this effect was regulated through PARP1.

Exosomes play the vital roles in cancer progression by transmitting their components, such as circRNAs, between cancer cells. Song et al.^30^ revealed that exosome‐transmitted circ_0000253 sponged miR141‐5p and downregulated SIRT1 to increase intervertebral disc degeneration. Another group reported that exosomal circ‐XIAP regulated the miR‐1182/TPD52 axis to promote PCa cell resistant to docetaxel.^39^ In our study, circTFDP2 was found to be enriched in the exosomes derived from PCa cells. Moreover, exosome‐delivered circTFDP2 promoted PCa cell progression. These results revealed that exosomal circTFDP2 is a promising target for PCa therapy.

Detection of serum PSA is the most common method for the diagnosis of PCa. However, the low specificity and accuracy of PSA test often cause the overdiagnosis of PCa.^40^, ^41^, ^42^ Thus, it is urgent to develop a novel diagnostic tool for the diagnosis of PCa. As we discussed before, noncoding RNAs, especially circRNAs, are enriched in exosomes. Thus, detection of exosomal circRNAs could be a useful way to diagnose PCa as a complement to PSA detection. Numerous studies have designed several biosensors to detect PCa‐related exosome noncoding RNAs.^40^, ^43^, ^44^ For instance, Kim et al.^40^ developed a 3D surface‐enhanced Raman scattering‐based biosensor that could detect an extensive range of concentrations of exosomal miRNAs (low to 10 aM) with high specificity. Therefore, exosomal circTFDP2 could also have the promising prospects for non‐invasive PCa diagnosis using novel biosensor technologies.

## 5 CONCLUSION

In summary, we discovered a novel PCa‐related circRNA, circTFDP2, which was upregulated in both PCa tissues and cell lines. circTFDP2 promoted PCa cell proliferation and metastasis by interacting with PARP1 and preventing it from caspase‐3‐dependent cleavage. The results also showed that the RBPs, eIF4A3, could regulate the biogenesis of circTFDP2. Moreover, exosome‐transmitted circTFDP2 promoted PCa tumorigenesis in vivo and in vitro (Figure 8I). Consequently, our study provides a promising therapeutic target for PCa.

## CONFLICT OF INTEREST

The authors declare they have no conflicts of interest.

## Supporting information

Supporting Information

Supporting Information

Supporting Information

Supporting Information

## Data Availability

The data that support the findings of this study are available from the corresponding author upon reasonable request.
