# Supplementary material for: Exosome‐derived circTFDP2 promotes prostate cancer progression by preventing PARP1 from caspase‐3‐dependent cleavage
Source: Clin Transl Med. 2023 Jan 3;13(1):e1156. doi: 10.1002/ctm2.1156 (PMC9810792; doi:10.1002/ctm2.1156)
Supplement: Supplementary file 1 — Supporting Information [file CTM2-13-e1156-s001.docx]

**Table S1**: Primers used for quantitative Real Time-PCR in this study.

| Gene Symbol | Forward primer (5’→3’) | Reverse primer (5’→3’) |
| --- | --- | --- |
| *circTFDP2* | *TGGTTTGACTTCCACAAATGC* | *AAACCTTCGTCTTCAATAATTC* |
| *TFDP2* | *CTGCCTACCAATTCTGCTCAG* | *CGCTTCTGCTTTATCCGTTCT* |
| *eIF4A3* | *AAGGGAGAGATGTCATCGCAC* | *GCTTGAGTTTCACGAACCTGA* |
| *PARP1* | *CGGAGTCTTCGGATAAGCTCT* | *TTTCCATCAAACATGGGCGAC* |
| *GAPDH* | *CAAGGTCATCCATGACAACTTTG* | *GTCCACCACCCTGTTGCTGTAG* |
| *circSEPT9* | *ttggagaattcagagcctg* | *gtcctggaatttctgggtggagc* |
| *CircASAP1* | *GCTAAAGAAAAGTGACGGTACAA* | *CAGCAGATCCACACCCTTTT* |

**Table S2**: siRNAs used in this study.

| Gene Symbol | siRNA sequence (sense 5’→3’) |
| --- | --- |
| *si-circTFDP2#2* | *GTCCAACAAAAGGTTGTTC* |
| *si-circTFDP2#3* | *CCAACAAAAGGTTGTTCTT* |
| *si-eIF4A3* | *AGAUCAAAGCUUGAGUUUCAC* |
| *si-PARP1#1* | *GAGCACUUCAUGAAAUUAUTT* |
| *si-PARP1#2* | *GAGGAAGGUAUCAACAAAUTT* |

**Table S3**: Antibody used in this study

| Antibody | company | catalog number |
| --- | --- | --- |
| *PARP1* | *cell signal technology* | *9532* |
| *β-actin* | *cell signal technology* | *3700* |
| *cleaved PARP1* | *cell signal technology* | *9548* |
| *BCL-2* | *cell signal technology* | *105071* |
| *Caspase-3* | *cell signal technology* | *9662* |
| *cleaved caspase-3* | *cell signal technology* | *9664* |
| *Bax* | *cell signal technology* | *5032* |
| *eIF4A3* | *Proteintech* | *7504-1-AP* |
| *Histone H3* | *cell signal technology* | *4499* |
| *yH2A.X* | *Huabio* | *et1602-2* |
| *GM130* | *Abcam* | *ab52649* |
| *Calnexin* | *Abcam* | *ab133615* |
| *HSP70* | *Abcam* | *ab2787* |
| *TSG101* | *Abcam* | *ab125011* |
| *CD9* | *Abcam* | *ab236630* |

**Table S4**: Biotinylated probe and sequence

| probe | sequence |
| --- | --- |
| *circTFDP2* | *5'biotin-aaaTCTTTAAAAGAACAACCTTTTGTTGGACTGAG* |
| *control probe* | *5' biotin-aaaGCAGCCTGATCACGACTGACTTTAGTGTTTGCATT* |
| *circTFDP2 segment 1* | *5' biotin-GTTGTTCTTTTAAAGAATTATTGAAGACGAAGGTTTTTTTCTTTTTATT* |
| *circTFDP2 segment 2* | *5' biotin-TTTTTAATGGCTTTACAGAATCTTAAATAGAATACAGTTTGACATGACGGCAA* |
| *circTFDP2 segment 3* | *5' biotin-AAATAGAATACAGTTTGACATGACGGCAAAAAATGTTGGTTTGACTTCCACA* |
| *circTFDP2 segment 4* | *5' biotin-GCAAAAAATGTTGGTTTGACTTCCACAAATGCAGAAGTAAGAGGATTTATAG*  *ATCAGAATCTCAGTCCAACAAAAG* |

**Table S5**: FISH oligonucleotide probe

| probe | sequence |
| --- | --- |
| *circPDE5A* | *5' CY3-* *AAAAGAACAACCT+TTTGT+TGGACTG 3’* |

**Table S6**: circTFDP2 specific binding proteins

| circTFDP2 specific binding proteins |
| --- |
| HNRPD |
| PGD |
| PRPF19 |
| RPL3 |
| ECH1 |
| HERPUD1 |
| SHMT2 |
| TUBB |
| HEL-S-30 |
| CTBP2 |
| FH |
| RPL29 |
| RPA1 |
| GPI |
| RPSA |
| PARP1 |
| HSDL2 |
| HSPB1 |
| TCP1 |
| ACAD9 |
| DARS1 |
| U2AF2 |
| TARS1 |
| SART3 |
| NDUFS2 |
| YBX1 |
