# Supplementary material for: Exosome‐derived circTFDP2 promotes prostate cancer progression by preventing PARP1 from caspase‐3‐dependent cleavage
Source: Clin Transl Med. 2023 Jan 3;13(1):e1156. doi: 10.1002/ctm2.1156 (PMC9810792; doi:10.1002/ctm2.1156)
Supplement: Supplementary file 3 — Supporting Information [file CTM2-13-e1156-s002.docx]

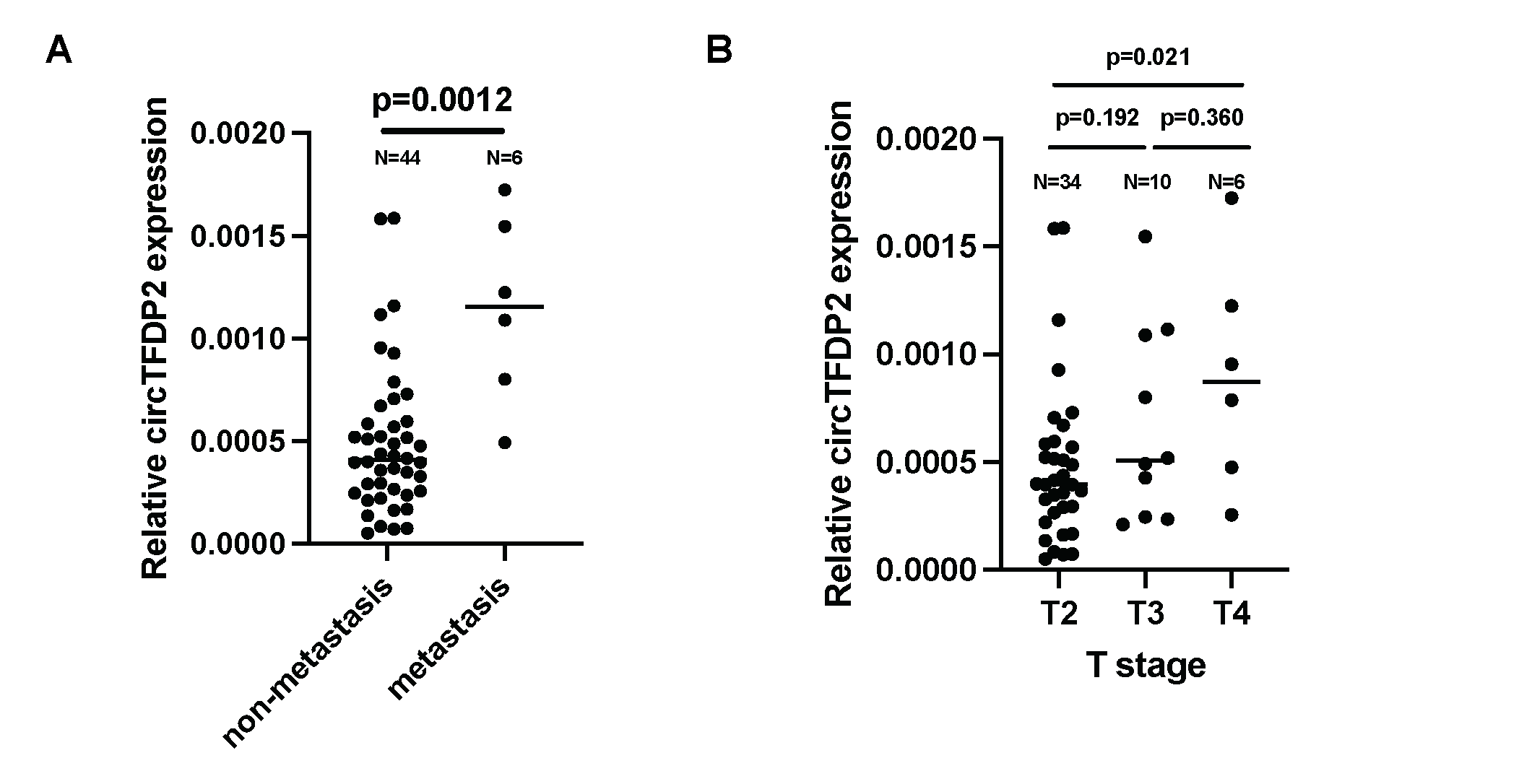


**Supplementary Figure 1,** **a,** Relative expression of circTFDP2 in 50 prostate cancer tissues with different metastatic status using qRT-PCR. **b,** Relative expression of circTFDP2 in 50 prostate cancer tissues with different T stage using qRT-PCR.


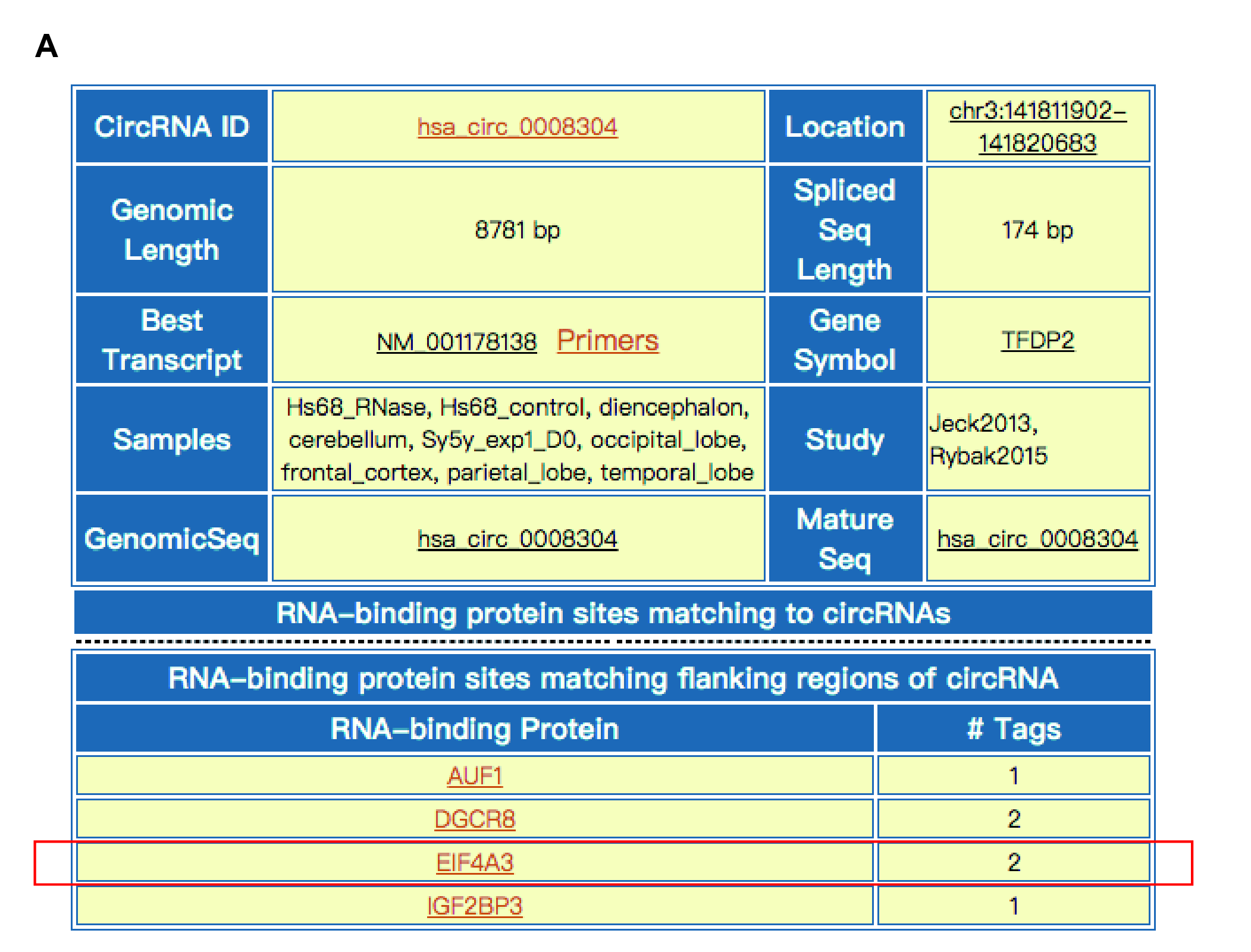


**Supplementary Figure 2,** **a,** The RNA-binding protein sites predicted by circinteractome database.

**
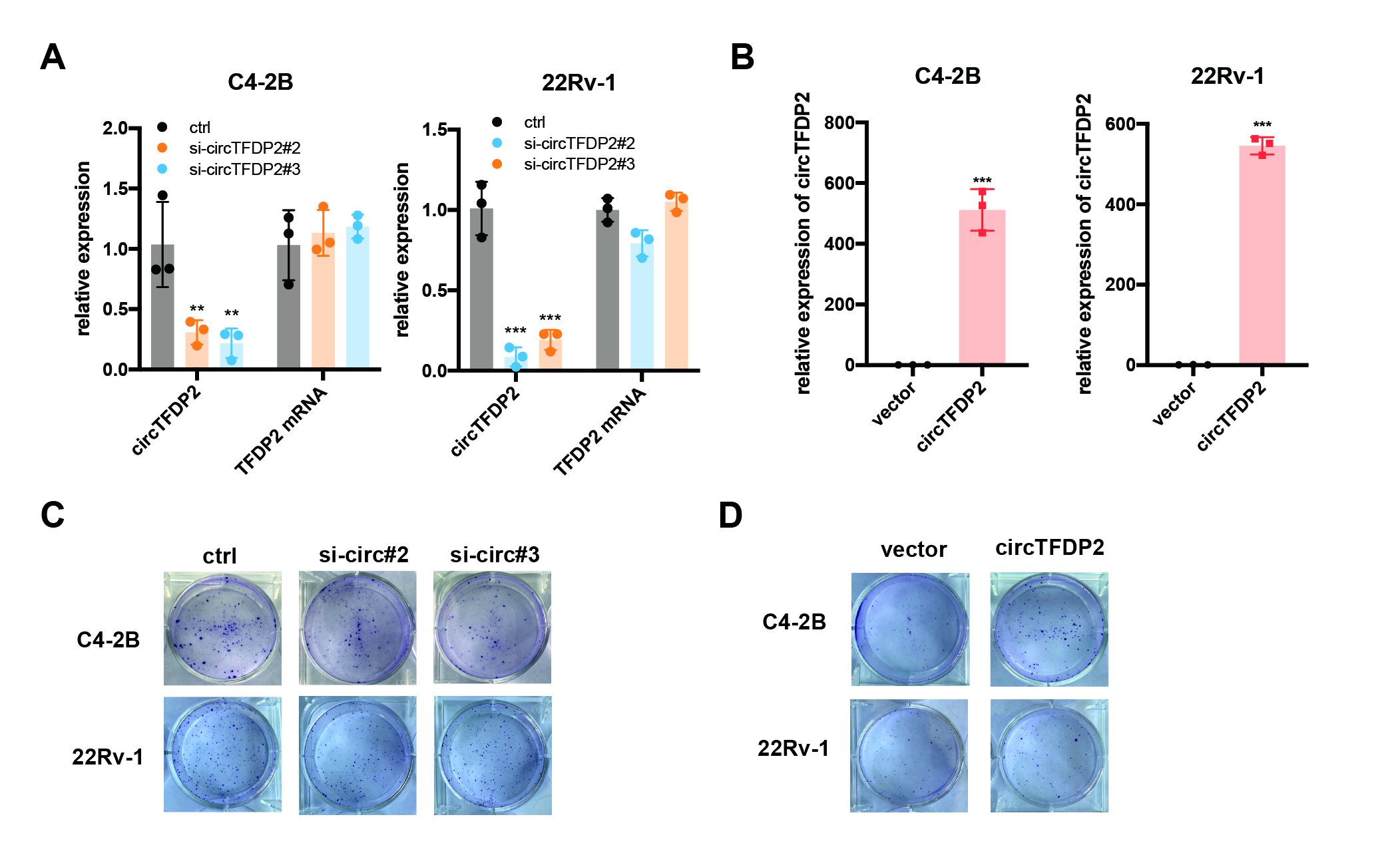
**

**Supplementary Figure 3,** **a,** Relative mRNA expression of circTFDP2 and TFDP2 with circTFDP2 knockdown. **b,** Relative expression of circTFDP2 with circTFDP2 overexpression. **c,** Colony formation assay for C4-2B and 22Rv-1 cells with circTFDP2 knockdown. **d,** Colony formation assay for C4-2B and 22Rv-1 cells with circTFDP2 overexpression.


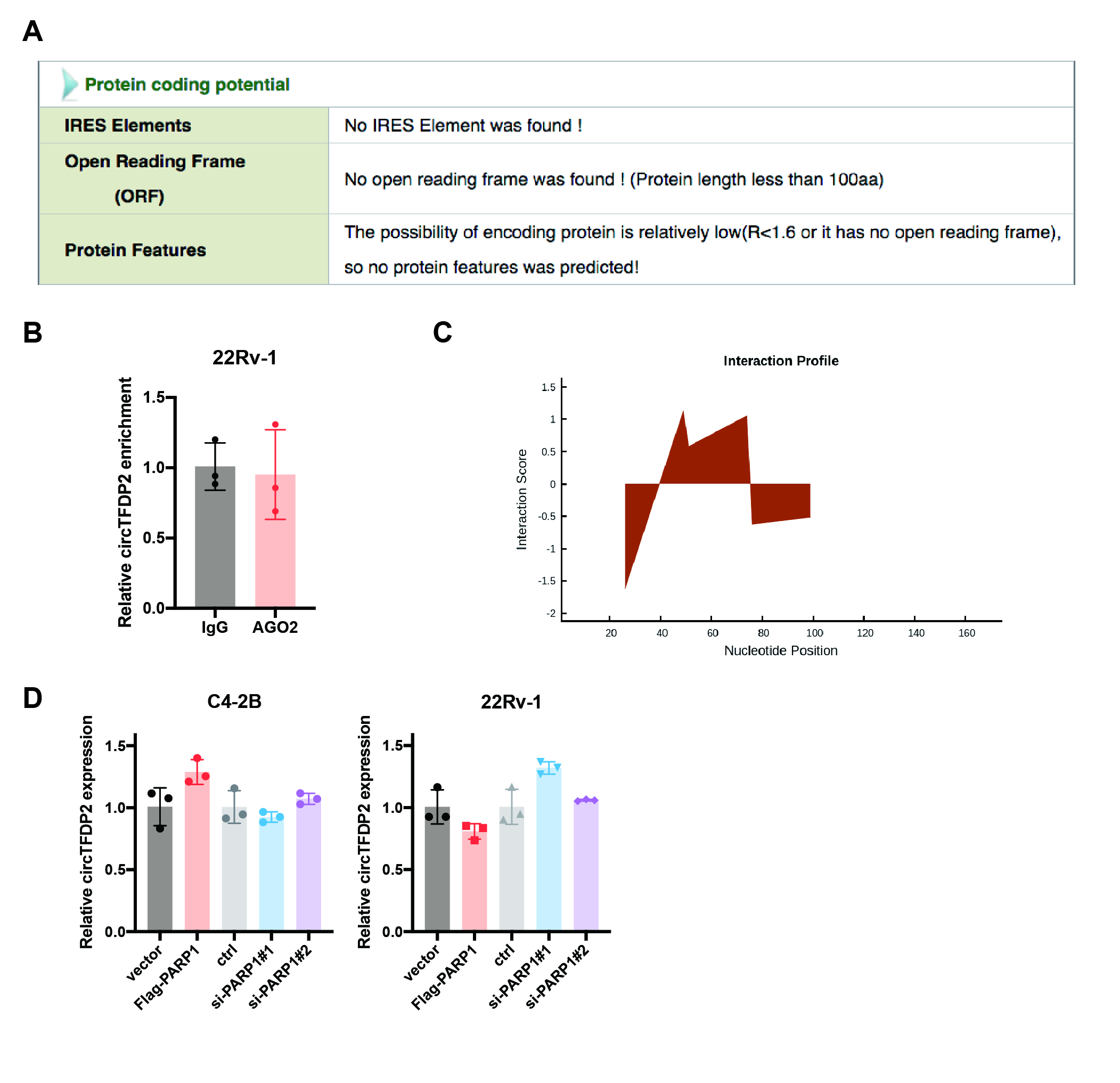


**Supplementary Figure 4,** **a,** The protein coding capacity of circTFDP2 predicted by circRNADb database. **b,** AGO2-RIP showing the interaction between AGO2 and circTFDP2. **c,** The interaction profile between circTFDP2 and PARP1 predicted by catRAPID algorithm. **d,** Relative expression of circTFDP2 with PARP1 knockdown or overexpression. Data represents mean±S.D. from three independent experiments. *, p < 0.05; **, p < 0.01; ***, p < 0.001.


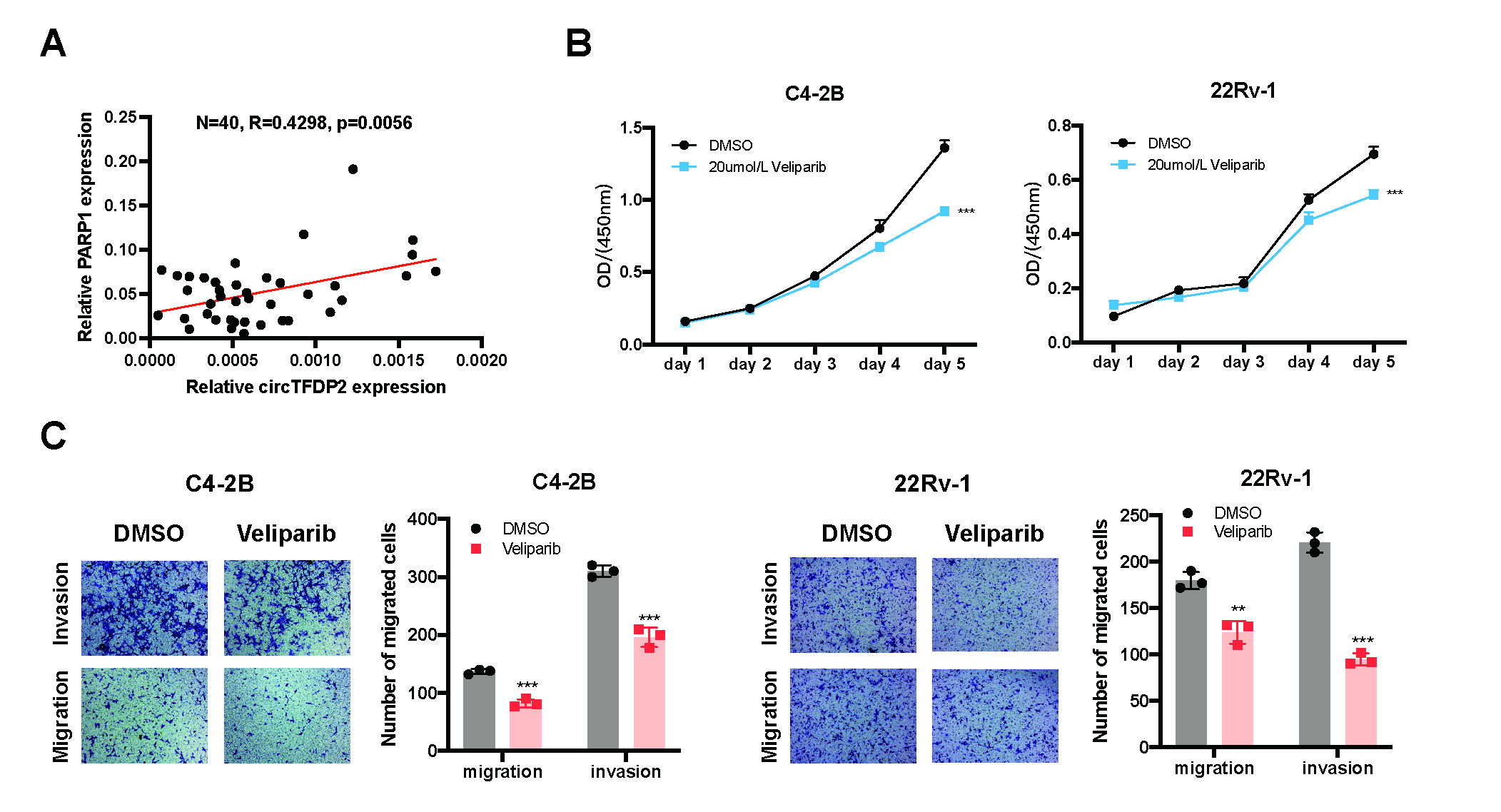


**Supplementary Figure 5, a,** Correlation analysis showed the positively correlation between circTFDP2 and PARP1 in 40 prostate cancer specimens. **b,** CCK-8 assay for C4-2B and 22Rv-1 cells treated with veliparib. **c,** Transwell assay for C4-2B and 22Rv-1 cells treated with veliparib. Data represents mean±S.D. from three independent experiments. *, p < 0.05; **, p < 0.01; ***, p < 0.001.


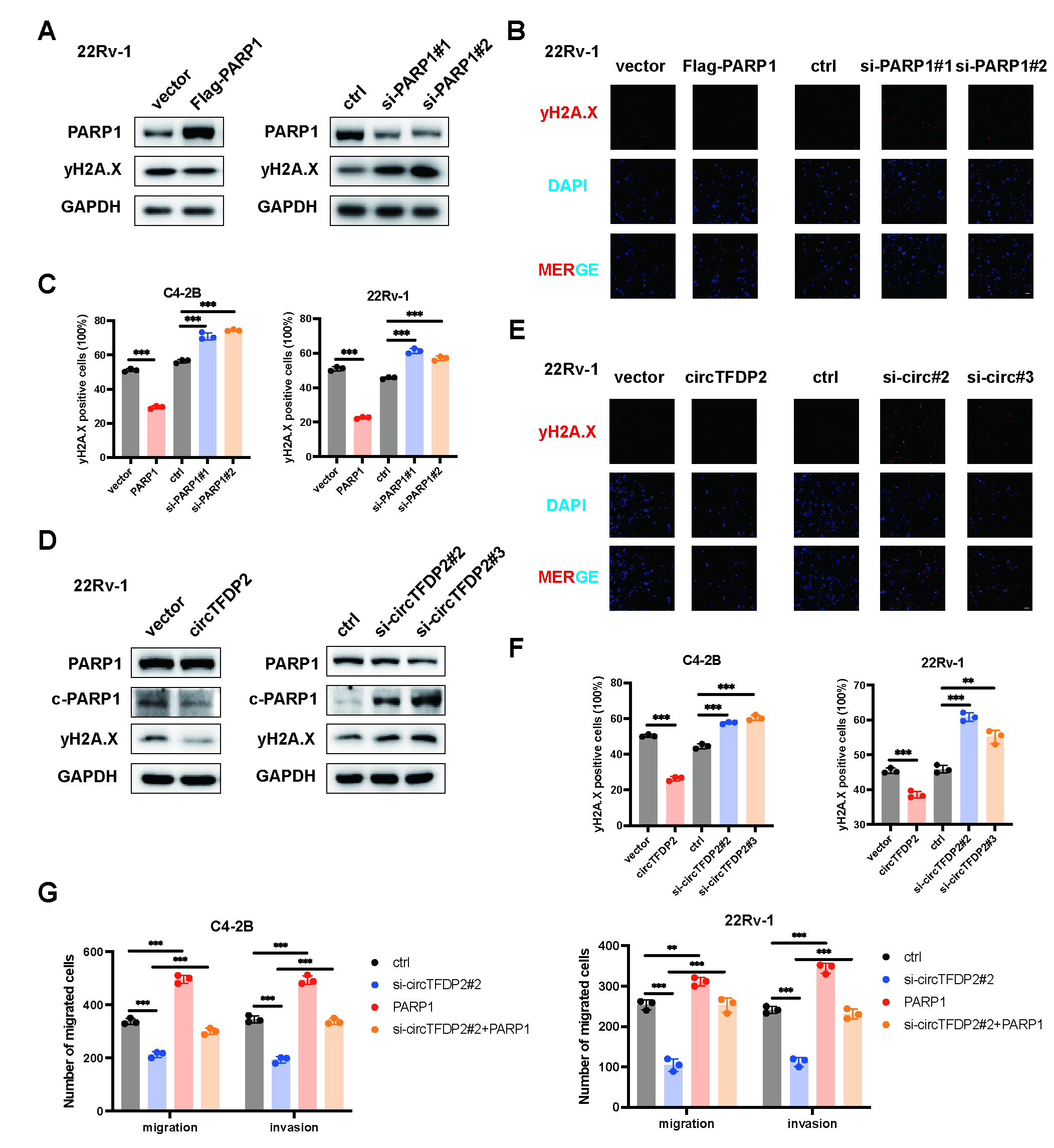


**Supplementary Figure 6, a,** Protein levels of DNA damage marker in PCa cells with PARP1 overexpression or knockdown. **b,** IF showing the yH2A.X expression with PARP1 overexpression or knockdown. scale bar = 100 um. **c,** Quantification of yH2A.X positive cell with PARP1 overexpression or knockdown. **d,** Protein levels of DNA damage marker in PCa cells with circTFDP2 overexpression or knockdown. **e,** IF showing the yH2A.X expression with circTFDP2 overexpression or knockdown. scale bar = 100 um. **f,** Quantification of yH2A.X positive cell with circTFDP2 overexpression or knockdown. **g,** Quantification of migrated cells in Transwell assay with circTFDP2 knockdown or PARP1 overexpression.
