# Supplementary material for: Exosome‐derived circTFDP2 promotes prostate cancer progression by preventing PARP1 from caspase‐3‐dependent cleavage
Source: Clin Transl Med. 2023 Jan 3;13(1):e1156. doi: 10.1002/ctm2.1156 (PMC9810792; doi:10.1002/ctm2.1156)
Supplement: Supplementary file 4 — Supporting Information [file CTM2-13-e1156-s003.docx]

**Materials and methods**

**Clinical samples**

All prostate cancer tissues were collected from the Zhejiang university School of Medicine, Sir Run Run Shaw Hospital. Informed consent was obtained from all patients. Furthermore, the Ethics Committee of Sir Run Run Shaw Hospital, School of Medicine, Zhejiang University approved this study. The detailed clinical information of patients was listed in the **Supplementary Table S7**.

**Cell culture**

Human prostate cancer cell lines LNCaP, DU145, PC-3, C4-2B, and 22Rv-1 and normal prostate epithelial cell line RWPE-1 were obtained from ATCC. All prostate cancer cells were cultured in RPMI 1640 medium (Gibco, USA) supplemented with 10% fetal bovine serum (FBS). Normal prostate epithelial cell RWPE-1 was cultured in K-SFM medium (Gibco, USA) supplemented with bovine pituitary extract and human epidermal growth factor. All cells were cultured at 37 °C with 5% CO_2_.

**RNA extraction and quantitative real-time PCR assays**

Total RNA was extracted using TRIzol reagent (CWbiotech, China) according to the manufacturer’s instructions. Then, 1000 ng RNA was used for reverse transcription with an All-in-One cDNA Synthesis SuperMix for PCR (Bimake, China). qRT-PCR was performed using 2× SYBR Green qPCR master mix (CWbiotech, China). The detailed primer sequences were listed in **Supplementary Table S1**.

**Plasmid and** **oligonucleotide transfection**

circTFDP2 siRNAs were synthesized by RiboBio (Guangzhou, China). eIF4A3 and PARP1 siRNAs were synthesized by GenePharma (Shanghai, China). All plasmids and circTFDP2 knockdown or overexpression lentiviruses were purchased from Geenchem (Shanghai, China). The transfection of siRNAs was performed by RNAimax (Invitrogen, USA). And plasmids were transfected into cells using Lipofectamine 3000 (Invitrogen, USA) according to the manufacturer’s instructions. Lentiviruses were used to construct the stably transfected PCa cell lines. After selection for puromycin resistance, stably transfected PCa cell lines were obtained. The siRNAs sequences used in this study were listed in **Supplementary Table S2**.

**Western blotting**

Western blotting was performed based on our previous study [1]. Total protein was extracted and separated using 8%-12% SDS-PAGE and transferred to PVDF membrane. Then, the membrane was incubated with the primary antibodies overnight at 4 °C, followed by incubation with corresponding secondary antibodies. The specific primary antibodies information was listed in **Supplementary Table S3**.

**Immunofluorescence**

Prostate cancer cells were cultured in 24-well plates with slides. The cells were fixed with 4% paraformaldehyde and permeabilized with 0.25% Triton X-100 for 10 min. After washing three times using 1xPBS, the cells were incubated overnight with yH2A.X antibody at 4 °C, then with the corresponding secondary antibody at room temperature. DAPI was used for nuclear staining. The images were photographed using the Olympus BX53 fluorescence microscope, and 10 X objective lens was applied.

**Cell proliferation and colony formation assays**

For cell proliferation assay, the transfected cells were plated onto the 96-well plate and cultured for 0, 24, 48, 72, and 96 hours. CCK-8 reagent (Dojindo Laboratories, Japan) was used to measure cell viability according to the manufacturer’s instruction.

For colony formation assay, the transfected cells were plated onto 6-well plate at a density of 500 cells/plate. After 14 days, colonies were fixed, and then stained with 0.1% crystal violet for 15 min.

**Transwell assay**

Migration and invasion assays were performed using transwell 8.0 um filters (Millipore, Germany) with or without Matrigel (BD Biosciences, USA) according to the manufacturer’s instructions. After 24 h, migrated cells through the membrane were fixed using 4% paraformaldehyde and stained with 0.1% crystal violet.

***In vivo* tumorigenesis and metastasis assay**

For xenograft animal model, 4-week-old BALB/c nude mice was used. 10^7^ circTFDP2 stably knockdown or overexpressing or negative control 22Rv-1 cells were subcutaneously injected into the BALB/c nude mice in the 100 μl 1xPBS. After 6-7 weeks, the tumors were harvested and the width (a) and length (b) of tumors were measured. The volume was calculated using the formula V = 1/2ab^2^.

For tail vein metastasis model, 10^7^ circTFDP2 stably knockdown or overexpressing or negative control 22Rv-1 cells were injected via the tail vein into the 4-week-old BALB/c nude mice. After 6-8 weeks, mice were anesthetized, following which the metastatic loci were photographed using *in vivo* imaging system (IVIS).

All procedures involving animals were approved by the Ethics Committee of Sir Run Run Shaw Hospital, School of Medicine, Zhejiang University.

**RNA pulldown assay**

The biotin-labelled circTFDP2 probe and four biotin-labelled circTFDP2 segment probes were synthesized by Tsingke (Beijing, China). 2x10^7^ 22Rv-1 and C4-2B cells were lysed with lysis buffer (50 mM Tris-HCl, pH 7.4, 150 mM NaCl, 2 mM MgCl_2_, 1% NP40, protease inhibitors, and RNase inhibitors) at 4 °C for 30 min. After centrifugation, the supernatants were incubated with according probe for 30 min at 4 °C, followed by incubation with 50 μl Streptavidin C1 magnetic beads (Invitrogen, USA). The beads were washed ten times with washing buffer (50 mM Tris-HCl, pH 7.4, 150 mM NaCl, 2 mM MgCl_2,_ and 1% NP40), and followed by Western blotting detection. The biotin-labeled circTFDP2 probe and four biotin-labelled circTFDP2 segment probes sequences were listed in **Supplementary Table S4**.

**Liquid chromatography–mass spectrometry analysis**

The proteins obtained from RNA pulldown assay were separated by SDS-PAGE. The gel was excised and digested to collect the eluents. Then, the peptides were separated using mobile phase A (100% water, 0.1% formic acid) and B solution (80% acetonitrile, 0.1% formic acid). The separated peptides were analyzed by Q Exactive^TM^ series mass spectrometer (Thermo Fisher). The MS data was searched against Uniprot database using proteome Discoverer 2.2 (PD 2.2, Thermo). Several criterions were applied to improve the quality of results: The identified protein contains at least 1 unique peptide. The FDR of identified Peptide Spectrum Matches (PSMs) and protein is no more than 1.0%.

**RNA** **Immunoprecipitation (RIP)**

The RNA-IP assay was performed using Magna RIP Kit (Millipore, USA) according to the manufactures’ instructions. Briefly, 5 μg AGO2, PARP1, or Flag antibodies was incubated with 50 μl magnetic beads at room temperature for 30 min to form antibody-beads complex. Then, 10^7^ C4-2B or 22Rv-1 cells were lysed with 500 μl IP buffer and incubated with antibody-beads complex at 4 °C overnight. After washing five times with washing buffer, the immunoprecipitated RNA was purified and detected by qRT-PCR analysis.

**RNA fluorescence in situ hybridization (FISH)**

The Cy3-labeled circTFDP2 probes were designed and synthesized by GenePharma (Shanghai, China). The FISH assay was performed using the FISH kit (GenePharma, China). The images were photographed using the fluorescence microscope (Leica, Germany). The probe sequence was listed in **Supplementary Table S5**.

**Isolation of exosomes from cell culture medium**

After incubation in the serum-free medium for 48 h, the medium was collected and centrifuged at 300 g and 3000 g for 30 minutes separately. Next, the supernatant was centrifuged at 100,000 g for 70 min. The pellet was suspended with 1 x PBS and filtered with 0.22-um filters. All steps were performed at 4 °C. For exosomes in vitro treatment assays, 1μg of exosomes (collected from approximately 5 x 10^6^ producer cells) were added to 3 x 10^6^ recipient cells.

**Transmission electron microscopy assay**

This assay was performed followed the method by Han et al. [2]. Briefly, the samples were washed in PBS three times, fixed in 1% osmium tetroxide for 60 min at room temperature. Then, these samples were fixed in glutaraldehyde and followed by dehydration with alcohol. Using propylene oxide to exchange pure alcohol. Samples were embedded in a pure, fresh Quetol-812 epoxy resin. Finally, the samples were observed and photographed using FEI Tecnai T20 transmission electron microscope.

**Statistical analysis**

Graphpad prism software v8.0 was used for analyses. Data was presented as means ± SD.

The data were analyzed using the t-test for two independent samples or one-way ANOVA followed by Student-Newman-Keuls test for more than two samples. The correlation analysis between eIF4A3 and circTFDP2 was examined by Pearson’s correlation test. All experiments were repeated three times. *P*-value less than 0.05 was considered significant. *, *p* < 0.05; **, *p* < 0.01; ***, *p* < 0.001.

1. Ding L, Wang R, Xu W, Shen D, Cheng S, Wang H, et al. PIWI-interacting RNA 57125 restrains clear cell renal cell carcinoma metastasis by downregulating CCL3 expression. Cell Death Discov [Internet]. 2021;7:333. Available from: https://doi.org/10.1038/s41420-021-00725-4

2. Zhang H, Deng T, Ge S, Liu Y, Bai M, Zhu K, et al. Exosome circRNA secreted from adipocytes promotes the growth of hepatocellular carcinoma by targeting deubiquitination-related USP7. Oncogene [Internet]. 2019;38:2844–59. Available from: https://doi.org/10.1038/s41388-018-0619-z
